# The apple 14-3-3 protein MdGRF11 interacts with the BTB protein MdBT2 to regulate nitrate deficiency-induced anthocyanin accumulation

**DOI:** 10.1038/s41438-020-00457-z

**Published:** 2021-02-01

**Authors:** Yi-Ran Ren, Qiang Zhao, Yu-Ying Yang, Tian-En Zhang, Xiao-Fei Wang, Chun-Xiang You, Yu-Jin Hao

**Affiliations:** 1 State Key Laboratory of Crop Biology, Shandong Collaborative Innovation Center for Fruit and Vegetable Production with High Quality and Efficiency, College of Horticulture Science and Engineering, Shandong Agricultural University, Tai-An, Shandong 271018 China; 2 Qingdao Agricultural University, Qingdao, Shandong 266109 China

**Keywords:** Abiotic, Translation, Plant signalling

## Abstract

Nitrogen is an important factor that affects plant anthocyanin accumulation. In apple, the nitrate-responsive BTB/TAZ protein MdBT2 negatively regulates anthocyanin biosynthesis. In this study, we found that MdBT2 undergoes posttranslational modifications in response to nitrate deficiency. Yeast two-hybrid, protein pull-down, and bimolecular fluorescence complementation (BiFC) assays showed that MdBT2 interacts with MdGRF11, a 14-3-3 protein; 14-3-3 proteins compose a family of highly conserved phosphopeptide-binding proteins involved in multiple physiological and biological processes. The interaction of MdGRF11 negatively regulated the stability of the MdBT2 protein via a 26S proteasome-dependent pathway, which increased the abundance of MdMYB1 proteins to activate the expression of anthocyanin biosynthesis-related genes. Taken together, the results demonstrate the critical role of 14-3-3 proteins in the regulation of nitrate deficiency-induced anthocyanin accumulation. Our results provide a novel avenue to elucidate the mechanism underlying the induction of anthocyanin biosynthesis in response to nitrate deficiency.

## Introduction

As sessile organisms, plants produce multiple secondary metabolites to cope with environmental stresses through sophisticated regulatory mechanisms^1^. Anthocyanins are a class of flavonoids ubiquitously distributed in plants. They are types of color-producing compounds in flowers, fruits, seeds, and leaves and provide plants with different colors—from orange-red to blue-purple^2,3^. For most plants, anthocyanin functions include resistance to low temperature, drought, and pathogen infections; protection against UV radiation and insect pests; attraction of pollinators; and facilitation of seed transmission^4–7^. Moreover, anthocyanins can benefit human nutrition and health due to their strong antioxidant properties and active oxygen-scavenging ability^8^.

It is well known that anthocyanins are derived from phenylpropanoids in the flavonoid biosynthesis pathway, in which a series of key enzymes and their encoding genes have been identified^9^. The expression of structural genes (*UF3GT*, *ANS*, *DFR*, *F3H*, *CHI*, and *CHS*) involved in anthocyanin biosynthesis is coordinately mediated by the MBW transcription complex, which is composed of three types of transcription factors (TFs): basic helix-loop-helix (bHLH), R2R3-MYB, and WD40-repeat proteins^10^. The MBW complex participates in the regulation of anthocyanin biosynthesis in various plant species^11^. In apple, the R2R3-MYB transcription factor MdMYB1 positively regulates anthocyanin biosynthesis by directly binding to the *MdANS* gene promoter^12^. The bHLH transcription factor MdbHLH3 promotes anthocyanin accumulation by activating the expression of *MdDFR* and *MdUFGT*^13^. In *Arabidopsis*, coexpression of the MYB TF *AtPAP1* with the bHLH TFs *AtTT8*, *AtGL3*, or *AtEGL3* can stimulate *AtDFR* promoter activity^14^.

Anthocyanin biosynthesis and accumulation are affected by endogenous signals such as phytohormones and sugar as well as by multiple environmental stimuli, including drought, low temperature, UV irradiation, intense light, wounding, and nutrient deficiency^15,16^. Nitrogen (N) is not only a necessary macronutrient for plant growth but also a key environmental factor that affects plant anthocyanin accumulation^17–19^. Nitrate deficiency induces the expression of bHLH and MYB TFs and represses the expression of zinc finger DNA-binding TFs *LBD37*, *38*, and *39* to promote anthocyanin biosynthesis in *Arabidopsis*^20,21^. Overexpression of *LBD37*, *38*, or *39* downregulates the expression of *PAP1* and *PAP2*, thereby inhibiting anthocyanin accumulation^21^. The RING-type E3 ubiquitin ligase NLA is involved in the induction of anthocyanin accumulation as an adaptative mechanisms to N limitation^22,23^. Furthermore, DELLA proteins have also been identified to be involved in nitrate deficiency-induced anthocyanin biosynthesis^24^.

Recently, the nitrate-responsive protein MdBT2 was identified to participate in the nitrate-mediated anthocyanin biosynthesis pathway^25^. BT2, a member of the BTB/TAZ family, has been identified as a central gene in the nitrogen-use efficiency (NUE) network^26,27^. The nitrate-responsive genes *NLP6* and *NLP7* transcriptionally activate *BT2* to mediate the nitrate response^28^. The apple and *Arabidopsis* genomes encode five BTB/TAZ proteins, all of which have a C-terminal calmodulin-binding domain, a central TAZ domain, and an N-terminal BTB domain^29,30^. In *Arabidopsis*, BT2 participates in multiple biological processes, including the regulation of gametophyte development and telomerase activity, as well as responses to light signals, hormones, stress, sugar, and nutrient status^31–33^. In apple, MdBT2 can interact with multiple types of substrate proteins to participate in different signaling pathways. For example, MdBT2 interacts with MdbHLH104, MdbHLH93, and MdMYB23 to negatively regulate Fe homeostasis, leaf senescence, and cold stress^30,34,35^.

Under adequate nitrate conditions, the expression of *MdBT2* is noticeably upregulated; in this case, MdBT2 mediates the ubiquitination and degradation of MdMYB1, thereby inhibiting anthocyanin accumulation^25^. However, the molecular mechanism underlying how MdBT2 is regulated in nitrate-deficiency conditions remains unclear. Posttranslational modification is a crucial regulatory mechanism through which plants regulate protein expression in response to multiple environmental stresses. Wounding, UV-B, high light, and drought induce MdBT2 ubiquitination^36–39^. Moreover, BT2 is predicted to be a potential target gene of two SnRK1 kinases, KIN10 and KIN11, based on microarray data^40^. In this study, we found that nitrate deficiency induced MdBT2 degradation via the 26S proteasome-dependent pathway. To further explore the underlying molecular mechanism, the MdBT2-interacting 14-3-3 protein MdGRF11 was identified by the Y2H method as a positive regulator of anthocyanin biosynthesis. MdGRF11 promoted the ubiquitination and degradation of the MdBT2 protein, which provided new insights for determining how anthocyanins accumulate under nitrate-deficiency conditions.

## Materials and methods

### Plant materials and treatments

Tissue cultures of apple GL-3, the seedlings of which were derived from open-pollinated Royal Gala (*Malus × domestica* cv. Royal Gala), were used as wild-type (WT) plants in this study. GL-3 tissue-cultured plantlets were grown on Murashige and Skoog (MS) media supplemented with 0.1 mg/L GA_3_, 0.2 mg/L NAA, and 0.3 mg/L 6-BA under a 16 h light/8 h dark photoperiod at 24 °C and subcultured every four weeks. The Orin apple calli used for genetic transformation were grown on MS media consisting of 1.5 mg/L 2,4-D and 0.4 mg/L 6-BA at normal temperature in darkness and subcultured at two-week intervals. Bagged apple fruits were harvested from Starkrimson apple trees at 120 days after full bloom (DAFB) for viral vector-mediated transient injection assays.

*Arabidopsis* ecotype Columbia (Col-0) was used for genetic transformation and phenotypic analysis. After being surface-sterilized and stratified for 3 days at 4 °C, *Arabidopsis* seeds were sown on 1/2-strength MS media under an 8/16-h dark/light photoperiod at 21 °C for 3 to 5 days and then treated with different concentrations of nitrate for determination of their anthocyanin contents. *grf9* (SALK_020026) mutants were obtained from the Arabidopsis Biological Resource Center (ABRC) mutant bank. The leaves of one-month-old *Nicotiana benthamiana* were used for bimolecular fluorescence complementation (BiFC) assays.

To detect the response of *MdBT2* and *MdGRF11* to nitrate deficiency, four-week-old apple (*Malus domestica* Rehd.) plantlets were treated with nitrogen-depleted Hoagland nutrient solution consisting of 5 mM KCl or 5 mM KNO_3_ for 3 days. Subsequently, the samples were used for qRT-PCR analysis.

For the phenotypic analysis of anthocyanin accumulation, the young shoots of four-week-old apple tissue culture GL-3 plantlets and two-week-old apple calli were transferred to the corresponding media deprived of nitrogen but consisting of either 5 mM KCl (nitrate deficiency) or 5 mM KNO_3_ (sufficient nitrate) under constant light for 7–10 days. The samples were subsequently harvested for expression analysis and measurement of anthocyanin contents.

### Vector construction and genetic transformation

The full-length CDSs of *MdBT2* and *MdGRF11* were inserted into a pCXSN-MYC vector to generate the *35* *S::MdBT2-OX* and *35* *S::MYC-MdGRF11* overexpression vectors. An antisense suppression expression vector *35* *S::MdBT2-Anti* was generated by cloning the specific fragment of *MdBT2* into a pCXSN vector. The 2000 bp promoter fragment of *MdGRF11* was cloned into a pCAMBIA1391-GUS vector, yielding a *Pro*_*MdGRF11*_*::GUS* recombinant construct. The resultant vectors *MdBT2-OX*, *MdBT2-Anti*, *MYC-MdGRF11*, and *Pro*_*MdGRF11*_*::GUS* were subsequently genetically transformed into Orin apple calli via the *Agrobacterium*-mediated transformation method^13^. The primers used for vector construction are listed in Supplementary Table 1.

*MdGRF11-OX* and *MdGRF11-Anti* transgenic calli and *MdGRF11* transgenic *Arabidopsis* were obtained as described previously^41^. Similarly, *MdBT2-GFP* transgenic calli were obtained as described previously^30^. *MdBT2-OX* and *MdBT2-Anti* transgenic apple plants were generated during our previous study^35^.

### Viral vector-mediated transient expression in apple skins and leaves

The viral vectors pIR and TRV were used for transient injection in apple^42,43^. The full-length cDNAs of *MdGRF11* and *MdBT2* were cloned into a pIR vector to generate pIR-MdGRF11 and pIR-MdBT2 overexpression viral vectors. Fragments of *MdGRF11* and *MdBT2* were inserted into a tobacco rattle virus (TRV) vector to generate the TRV-MdGRF11 and TRV-MdBT2 suppression viral vectors. Moreover, the suppression viral vectors and an empty TRV vector were transformed into *A. tumefaciens* strain LBA4404. The recombinant plasmids or the *A. tumefaciens* solutions were subsequently injected into fresh-bagged apple fruit peels and apple leaves. The auxiliary vectors IL-60-BS and TRV1 were used for all sense or antisense coinjections, respectively. Fruit and leaf injection assays were performed as previously described^44,45^. pIR and TRV empty vectors were used as controls.

The injected apples were kept in darkness at room temperature overnight and subsequently exposed to continuous white light at 21 °C for 4–6 days for coloration. The injected apple leaves were placed in 5 mM KCl-treated media under constant light at 21 °C for 5 d.

### Gene expression analysis

Total RNA of apple calli, leaves, and fruit peels was extracted with an RNAplant Extraction Kit (CWBIO, Beijing, China), and cDNA was synthesized from one to five micrograms of RNA using a PrimeScript RT reagent kit (Takara, Dalian, China). qRT-PCR was performed with Ultra SYBR Mixture (CWBIO) in conjunction with an ABI 7500 Real-Time PCR System (Applied Biosystems) as described previously^46^. The relative gene expression level was calculated with the 2^−ΔΔCT^ (Ct) method. The *18s* gene was used as an internal reference. All samples were analyzed as three biological replicates. Sequences of the primers used for gene expression are listed in Supplementary Table 1.

### Protein extraction and Western blot analysis

Apple calli or plantlets were ground into powder in liquid nitrogen and immediately immersed in protein extraction buffer [100 mM NaCl, 0.5 mM EDTA, 20 mM Tris-HCl (pH 7.5), 0.5 mM PMSF, 0.5% Nonidet P-40 and 0.5% protease inhibitor] for 15 min on ice. The mixture was then centrifuged at 12,000 rpm for 15 min at 4 °C. The total protein supernatant was collected and separated on a 10% SDS-PAGE gel, transferred to a polyvinylidene fluoride (PVDF) membrane (Roche, IN, USA), and probed with anti-GFP antibody (Abmart, Shanghai, China). Western blotting assays were conducted as previously described^30^. ACTIN served as a protein loading control.

### Y2H screening and assays

To screen the proteins that interact with MdBT2, the full-length cDNA of *MdBT2* was fused to the binding domain of the pGBT9 vector as bait (BD-MdBT2). The apple cDNA library was generated from apple skin and constructed by Oebiotech Company. Y2H screening was conducted according to the Yeast Transformation System 2 manufacturer’s instructions (Clontech, Shanghai, China), and the positive clones were determined by sequencing.

For the Y2H assays, the coding sequences of *AtBTs* and *MdBTs* and the truncated sequences of *MdBT2* were cloned into a pGBT9 bait vector, while the full-length CDSs of *MdGRFs* and *AtGRFs* were inserted into a pGAD424 prey vector. The recombinant constructs were then cotransformed into yeast strain Y2H Gold as described in the manufacturer’s instructions (Clontech). The transformed yeast cells were cultured on SD/-Trp-Leu (-T/-L) and SD/-Trp-Leu-His-Ade (-T/-L/-H/-A) media supplemented with 5-bromo-4-chloro-3-indolyl α-D-galactopyranoside (X-α-gal) for selection.

### Pull-down assays

The full-length cDNA of *MdBT2* was inserted into a PGEX-4T-1 vector for glutathione S-transferase (GST) tag fusion, and the coding sequence of *MdGRF11* was cloned into a PET-32a vector for His_6_ tag fusion. Both recombinant vectors were transformed into *Escherichia coli* BL21 for the expression of GST-MdBT2 and His-MdRGL3a proteins via 0.1 mM isopropyl β-D-1- thiogalactopyranoside (IPTG) induction, respectively. Subsequently, the protein mixtures containing equal quantities of GST-MdBT2 and His-MdRGL3a proteins were eluted using a Pierce GST Spin Purification Kit (Thermo Fisher Scientific, MA, USA). GST protein acted as a negative control. The eluted solution was then subjected to immunoblot analysis with anti-HIS and anti-GST antibodies (Abmart).

### BiFC assays

pSPYNE-35S, pSPYCE-35S, and the 35 S:p19 cotransformation vector were used for BiFC assays as described previously^47^. The full-length cDNAs of *MdBT2* and *MdGRF11* were inserted into pSPYNE-35S and pSPYCE-35S, which contain DNA sequences encoding the N- or C-terminal halves of YFP (YFP^N^ or YFP^C^), respectively. The recombinant constructs MdBT2-YFP^N^ and MdGRF11-YFP^C^ were subsequently introduced into *Agrobacterium* strain GV3101. The *Agrobacterium* strains containing different combinations and the *Agrobacterium* carrying the p19 silencing plasmid were incubated and cosuspended in infiltration buffer consisting of 10 mM MgCl_2_, 10 mM MES, and 0.2 mM acetosyringone to a final concentration of which the OD600 was 0.5. YFP^N^ and YFP^C^ empty vectors were used as negative controls. Subsequently, *N. benthamiana* leaf epidermal cells were infiltrated and incubated at 24 °C for 2 days. YFP fluorescence was detected using a Zeiss LSM 510 Meta high-resolution laser-scanning confocal microscope (Jena, Germany).

### Cell-free and in vivo protein degradation assays

For cell-free degradation assays, total proteins of apple calli or plantlets were extracted with protein degradation buffer comprising 25 mM Tris-HCl (pH 7.5), 10 mM MgCl_2_, 5 mM DTT, 10 mM NaCl, 4 mM PMSF, and 10 mM ATP, as previously described^46^. Afterward, 100 ng of *Escherichia coli* BL21-induced GST-MdBT2, HIS-MdMYB1, and HIS-MdGRF11 recombinant proteins and equal amounts of the above total proteins (500 μg) were incubated together at 22 °C for different time periods. The samples were then subjected to immunoblot analysis with anti-GST or anti-His antibodies. For the proteasome inhibitor MG132 treatment, total protein extracts were pretreated with 50 μM MG132 for 30 min before being incubated together with recombinant proteins.

In nitrate-mediated GST-MdBT2 protein stability assays, four-week-old apple plantlets (*Malus domestica* Rehd.) were pretreated with nitrogen-depleted Hoagland nutrient solution consisting of either 5 mM KCl or 5 mM KNO_3_ for 3 days, after which the total protein extracts from these plantlets and recombinant GST-MdBT2 proteins were incubated together for the indicated times. The proteins were detected using anti-GST antibodies.

For in vivo degradation assays, two-week-old *MdBT2-GFP* and *MYC-MdGRF11* + *MdBT2-GFP* calli were treated with 250 μM cycloheximide (CHX) with or without 50 μM MG132 in the dark. Total proteins were extracted at the indicated times with the abovementioned protein extraction buffer and then analyzed by immunoblotting using an anti-GFP antibody. Quantification of the protein levels was performed by Quantity One software (Bio-Rad, CA, USA), and ACTIN was used as a control.

### Ubiquitination assays

For in vivo ubiquitination assays, *MYC-MdBT2* and *MdGRF11-GFP* + *MYC-MdBT2* calli were pretreated with 50 μM MG132 for 6 h in the dark before extraction. The MYC-MdBT2 proteins were immunoprecipitated with an anti-MYC antibody (Abmart) according to the method for the Pierce Classic Protein A IP Kit (Thermo Fisher Scientific). The resultant proteins were subjected to immunoblot analysis with anti-MYC and anti-ubiquitin antibodies (Sigma-Aldrich, MO, USA).

### Determination of anthocyanin contents

Total anthocyanins in apple tissue culture GL-3 plantlets, leaves, fruit peels, and calli and in *Arabidopsis* were extracted via the methanol-HCl method. The plant materials were incubated in anthocyanin extraction solution [1% (v/v) methanol-HCl] in darkness at room temperature for 24 h. The absorbance values of the extract samples were determined at 530, 620, and 650 nm with a spectrophotometer (SOPTOP, Shanghai, China). The relative anthocyanin content was calculated according to previously described methods^48^.

### GUS staining and activity analysis

For histochemical staining, *Pro*_*MdGRF11*_*::GUS* calli treated with 5 mM KCl or 5 mM KNO_3_ were incubated in GUS staining buffer (1 mM 5-bromo-4-chloro-3-indolyl-β-glucuronide, 10 mM EDTA, 0.1% Triton X-100, 0.05 mM ferricyanide, 0.05 mM ferrocyanide, 20% methanol and 0.1 M sodium phosphate buffer, pH 7.0) at 37 °C for 12 h.

GUS activity analysis was carried out according to previously described methods^13^.

### Accession numbers

Sequence data of the following genes (and their accession numbers) in this article can be found in the GDR: MdBT1 (MDP0000151000), MdBT2 (MDP0000643281), MdBT3.1 (MDP0000296225), MdBT3.2 (MDP0000187156), MdBT4 (MDP0000215415), MdGRF11 (MD10G1280300), MdGRF4 (MD05G1301400), MdGRF6 (MD07G1096000), and MdGRF18 (MD17G1074900). Similarly, sequence data of the following genes (and their accession numbers) in this article can be found in the Arabidopsis Genome Initiative database: AtBT1 (At5g63160), AtBT2 (At3g48360), AtBT3 (At1g05690), AtBT4 (At5g67480), AtBT5 (At4g37610), AtGRF1 (At4g09000), AtGRF2 (At1g78300), AtGRF3 (At5g38480), AtGRF4 (At1g35160), AtGRF5 (At5g16050), AtGRF6 (At5g10450), AtGRF7 (At3g02520), AtGRF8 (At5g65430), AtGRF9 (At2g42590), AtGRF10 (At1g22300), AtGRF11 (At1g34760), AtGRF12 (At1g26480), and AtGRF13 (At1g78220).

## Results

### Nitrate deficiency negatively regulates MdBT2 expression

**Fig. 1:**
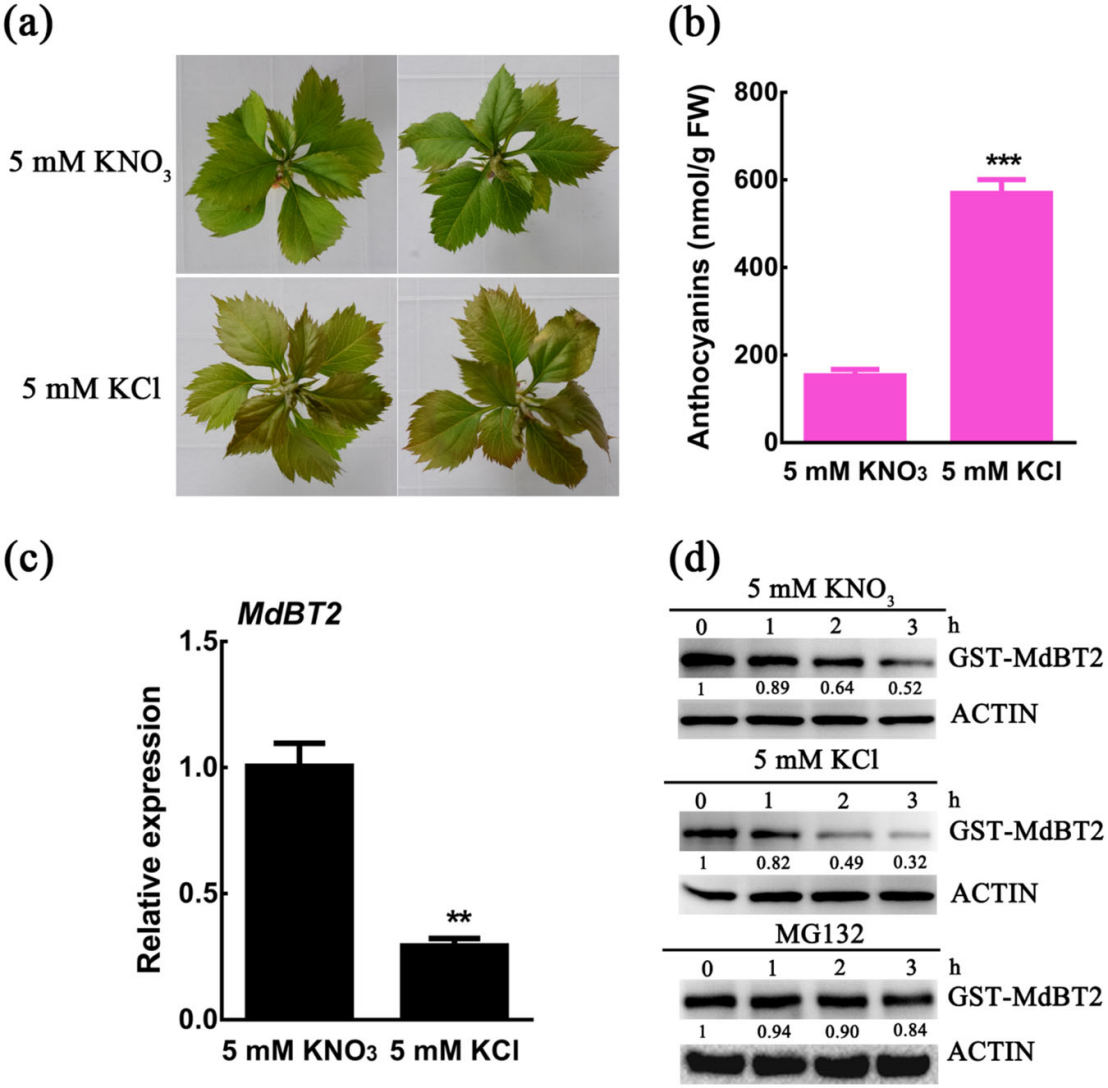
Nitrate deficiency negatively regulates MdBT2 expression. **a** Anthocyanin pigmentation phenotype of four-week-old apple tissue culture GL-3 plantlets treated with 5 mM KCl (nitrate deficiency) or 5 mM KNO_3_ (sufficient nitrate) under constant light for 10 d. **b** Anthocyanin content in apple tissue culture GL-3 plantlets treated with 5 mM KCl or 5 mM KNO_3_. The error bars indicate the SDs of three biological replicates, with at least 6 plantlets per replicate. The asterisks indicate significant differences (***P < 0.001) based on Student’s *t* test. **c** Expression analysis of *MdBT2* in response to nitrate deficiency. Four-week-old apple plantlets were treated with nitrogen-depleted Hoagland nutrient solution supplemented with 5 mM KCl or 5 mM KNO_3_ for 3 days. The *18s* gene was u*s*ed as an internal reference. The error bars indicate the SDs of three independent biological replicates. The asterisks indicate significant differences (**P < 0.01) based on Student’s *t* test. **d** Cell-free degradation assays of MdBT2 in response to nitrate treatment. Four-week-old apple plantlets were treated with 5 mM KCl or 5 mM KNO3 for 3 days, and then the total protein extracts from these plantlets and recombinant GST-MdBT2 proteins were incubated together for the indicated times. For the proteasome inhibitor MG132 treatment, total protein samples extracted from 5 mM KCl-treated apple plantlets were pretreated with 50 μM MG132 for 0.5 h before sampling began. Anti-GST antibody was used for immunoblotting. ACTIN was used as loading control. The protein levels at 0 h were set to 1

Nitrate deficiency induces anthocyanin accumulation in apple^49^. The young shoots of apple tissue culture GL-3 plantlets treated with KCl (nitrate deficiency) accumulated higher levels of anthocyanin content than the young shoots of KNO_3_-treated (sufficient nitrate) GL-3 plantlets did (Fig. 1a, b). MdBT2 has been shown to be a negative regulator of anthocyanin accumulation^25^. Here, we specifically detected the effect of MdBT2 on anthocyanin biosynthesis under nitrate-deficiency conditions. After KCl treatment, compared with the wild-type (WT) controls, the *MdBT2* overexpression transgenic apple lines (*MdBT2-OX1*, *OX5*, and *OX7*) accumulated less anthocyanin, while suppression lines (*MdBT2-Anti13* and *Anti23*) accumulated more anthocyanin (Supplementary Fig. 1a–c). Furthermore, the expression levels of genes related to anthocyanin biosynthesis, including *MdDFR*, *MdANS*, and *MdUF3GT*, were markedly downregulated in the *MdBT2* overexpression transgenic apple lines (Supplementary Fig. 1d–f).

To further study the molecular mechanism of MdBT2 in response to nitrate deficiency regulating anthocyanin biosynthesis, the transcriptional and posttranslational levels of MdBT2 were measured in response to nitrate deficiency. qRT-PCR assays showed that the transcript level of *MdBT2* was noticeably downregulated after treatment with KCl (Fig. 1c). In addition, a cell-free protein degradation assay was also conducted to detect the abundance of the MdBT2 protein. The immunoblot results showed that, compared with KNO_3_ treatment, KCl treatment accelerated GST-MdBT2 protein degradation (Fig. 1d). Moreover, the degradation of MdBT2 was significantly inhibited in the presence of the proteasome inhibitor MG132 (Fig. 1d), indicating that the degradation of MdBT2 was dependent on the 26S proteasome pathway. Taken together, these findings indicated that nitrate deficiency suppressed the expression of MdBT2 at the transcriptional and posttranslational levels.

### MdGRF11 interacts with MdBT2

**Fig. 2:**
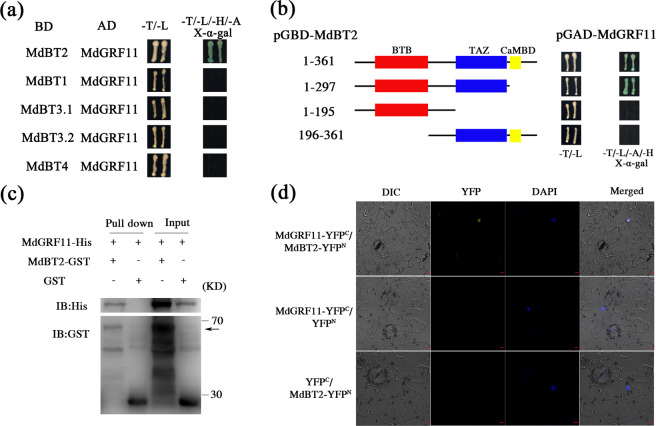
MdGRF11 interacts with MdBT2 proteins. **a** Yeast two-hybrid assay to examine the interactions of MdGRF11 with MdBT1, MdBT2, MdBT3.1, MdBT3.2, and MdBT4. Yeast cells were grown on SD/-Trp-Leu (-T/-L) and SD/-Trp-Leu-His-Ade (-T/-L/-H/-A) media. X-α-gal was used to further confirm the positive interactions. **b** Y2H assay to determine the domain through which MdBT2 interacts with MdGRF11. **c** In vitro pull-down assay indicating the interaction of MdBT2 and MdGRF11. His-MdGRF11 proteins and GST-MdBT2 or GST fusion proteins were incubated together and then purified with a GST column. Immunoprecipitated proteins were detected with anti-HIS and anti-GST antibodies. IB immunoblotted. **d** Interaction between MdBT2 and MdGRF11 detected by a BiFC assay in transiently transformed *N. benthamiana* leaf epidermal cells. DAPI was used as a marker for the nucleus. YFP fluorescence was observed via confocal microscopy. Scale bars, 10 μm

A Y2H screening assay was carried out to identify the potential proteins mediating the stability of MdBT2 in response to nitrate deficiency. The CDS of *MdBT2* was integrated into the binding domain of a pGBT9 vector as bait (BD-MdBT2) to screen its interacting partners in the apple cDNA library. After screening, a positive colony that contained a cDNA fragment encoding the 14-3-3 protein MD10G1280300, named MdGRF11, was isolated^41^. To verify the interaction, the ORF sequence of *MdGRF11* was fused to a pGAD424 vector as prey (AD-MdGRF11) and cotransformed into yeast together with BD-MdBT2 for a Y2H assay. MdGRF11 interacted with MdBT2 (Fig. 2a). Furthermore, the interactions of MdGRF11 with the other four MdBTs were also investigated. However, MdGRF11 interacted only with MdBT2 and not with MdBT1, MdBT3.1, MdBT3.2, or MdBT4 (Fig. 2a). Previous studies have indicated that MdGRF11 can form a heterodimer together with MdGRF4, MdGRF6, and MdGRF18^41^. The Y2H assay showed that MdBT2 also interacted with MdGRF4, MdGRF6, and MdGRF18 (Supplementary Fig. 2a). The *Arabidopsis* AtBT2 protein is homologous to MdBT2^25^, and the Y2H assay showed that AtBT2 also interacted with MdGRF11, AtGRF8, AtGRF9, and AtGRF10 proteins (Supplementary Fig. 2b, c). To determine which domain of MdBT2 was required for interacting with MdGRF11, MdBT2 was divided into its C-terminal calmodulin-binding domain, TAZ domain, and N-terminal BTB domain. As shown in Fig. 2b, both the BTB and TAZ domains are required for MdBT2 interaction with MdGRF11 (Fig. 2b).

The in vitro interaction between MdBT2 and MdGRF11 was confirmed by a pull-down assay. The results showed that the His-MdGRF11 protein was pulled down only by GST-MdBT2 and not by the GST control (Fig. 2c), demonstrating that MdGRF11 interacted with MdBT2 in vitro. In addition, a BiFC assay was carried out to further determine their interactions in vivo. MdGRF11 and MdBT2 were integrated into the C-terminal fragment or N-terminal fragment of YFP, yielding MdGRF11-YFP^C^ and MdBT2-YFP^N^ constructs, respectively. As a result, a strong yellow fluorescent signal was detected in the nucleus of epidermal cells cotransformed together with MdGRF11-YFP^C^ and MdBT2-YFP^N^. In contrast, none of the fluorescent signals were observed in the control samples (Fig. 2d). These results indicated that MdGRF11 physically interacted with MdBT2.

### Nitrate deficiency positively regulates MdGRF11 expression

**Fig. 3:**
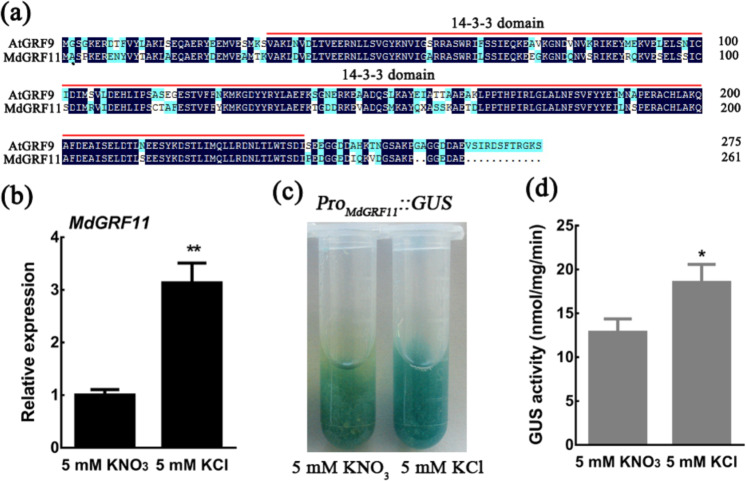
Nitrate deficiency positively regulates MdGRF11 expression. **a** Protein sequence alignment of MdGRF11 and its orthologous protein AtGRF9. The red line represents the scope of the conserved 14-3-3 domain. **b** qRT-PCR analysis of the relative *MdGRF11* expression level in response to nitrate deficiency. Four-week-old apple plantlets were treated with 5 mM KCl or 5 mM KNO_3_ for 3 days. The *18s* gene was used as an internal reference. **c** GUS staining images of *Pro*_*MdGRF11*_*::GUS* transgenic calli treated with 5 mM KCl or 5 mM KNO_3_ for 12 h. **d** Quantitative analysis of GUS activity in **c**. In **b**, **d**, the error bars indicate the SDs of three independent experiments. The asterisks indicate significant differences (*P < 0.05; **P < 0.01) based on Student’s *t* test

14-3-3 phosphopeptide-binding proteins are highly conserved in cells of eukaryotes, including plants, animals, and yeast^50^. Protein sequence alignment showed that MdGRF11 and its orthologous protein AtGRF9 contained a conserved 14-3-3 domain (Fig. 3a). To explore the role of MdGRF11 in the response to nitrate deficiency, the expression levels of *MdGRF11* in response to KCl and KNO_3_ were measured. The results showed that nitrate deficiency (KCl treatment) significantly induced the expression of *MdGRF11* (Fig. 3b). Furthermore, a GUS staining assay showed that the KCl treatment caused much higher GUS activity than did the KNO_3_ treatment in *Pro*_*MdGRF11*_*::GUS* transgenic calli, which was opposite the expression pattern of *MdBT2* (Fig. 3c, d). Taken together, these results indicated that MdGRF11 played a positive role in the response to nitrate deficiency.

### MdGRF11 promotes nitrate deficiency-induced anthocyanin accumulation

**Fig. 4:**
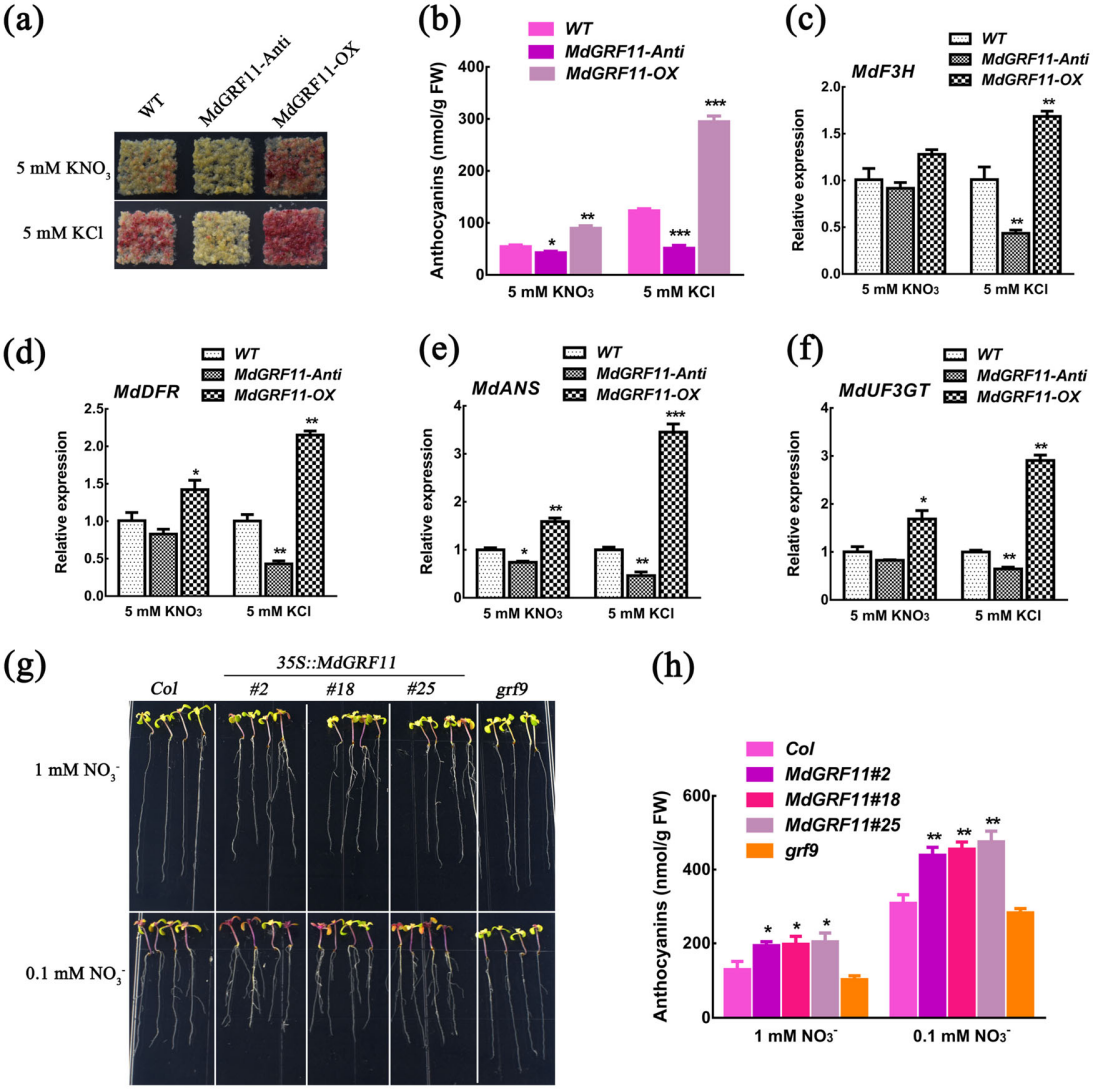
MdGRF11 promotes nitrate deficiency-induced anthocyanin accumulation. **a** Callus coloration of two-week-old wild-type (WT), *MdGRF11-OX*, and *MdGRF11-Anti* transgenic calli treated with 5 mM KCl or 5 mM KNO_3_ for 7 days. **b** Anthocyanin contents in **a**. **c**–**f** Expression analysis via qRT-PCR of the anthocyanin biosynthesis-related genes *MdF3H*, *MdDFR*, *MdANS*, and *MdUF3GT* in the apple calli shown in **a**. The *18s* gene acted as an internal control. **g** Phenotypes of Col-0, *grf9* mutant, and *MdGRF11* transgenic *Arabidopsis* seedlings (*35* *S::MdGRF11#2*, #18 and #25) grown on media consisting of 0.1 mM KNO_3_ or 1 mM KNO_3_. **h** Anthocyanin contents in **g**. In **b**–**f**, **h** the error bars indicate the SDs of three independent experiments, each of which included three technical replicates. The asterisks indicate significant differences (*P < 0.5; **P < 0.01; ***P < 0.001) based on Student’s *t* test

Considering that MdGRF11 interacted with MdBT2 and participated in the response to nitrate deficiency, it was hypothesized that MdGRF11 is involved in nitrate-mediated anthocyanin accumulation. To prove this hypothesis, Orin apple calli (WT), *MdGRF11* overexpression (*MdGRF11-OX*), and *MdGRF11* suppression (*MdGRF11-Anti*) transgenic calli were used to detect anthocyanin accumulation. In *MdGRF11-Anti* transgenic calli, the expression levels of *MdGRF4*, *MdGRF6*, and *MdGRF18* were also inhibited, possibly due to sequence similarity and functional redundancy (Supplementary Fig. 3a). After treatment for 7 days, three types of calli produced less anthocyanins in response to KNO_3_ than to KCl (Fig. 4a, b). Moreover, compared with the WT control calli, *MdGRF11-OX* transgenic calli noticeably accumulated more anthocyanins, and *MdGRF11-Anti* transgenic calli accumulated less anthocyanins, particularly in the media treated with KCl (Fig. 4a, b). In accordance with the anthocyanin phenotypes, the expression of the anthocyanin biosynthesis-related genes *MdF3H*, *MdDFR*, *MdANS*, and *MdUF3GT* was markedly upregulated in the *MdGRF11-OX* transgenic calli but suppressed in the *MdGRF11-Anti* transgenic calli (Fig. 4c–f).

*MdGRF11* transgenic *Arabidopsis* was also generated to confirm the function of MdGRF11 in regulating anthocyanin accumulation. Nitrate treatment promoted root growth and inhibited anthocyanin accumulation in *Arabidopsis* (Supplementary Fig. 4). Overexpression of *MdGRF11* also promoted nitrate deficiency-induced anthocyanin biosynthesis (Fig. 4g, h). However, the phenotype of the *grf9* mutant did not differ from that of the control (Col-0) (Fig. 4g, h and Supplementary Fig. 3b), indicating that functional redundancy exists among GRF members. In conclusion, these data suggested that MdGRF11 acted as a positive regulator of anthocyanin accumulation induced by nitrate deficiency.

### MdGRF11 promotes the destabilization of MdBT2

**Fig. 5:**
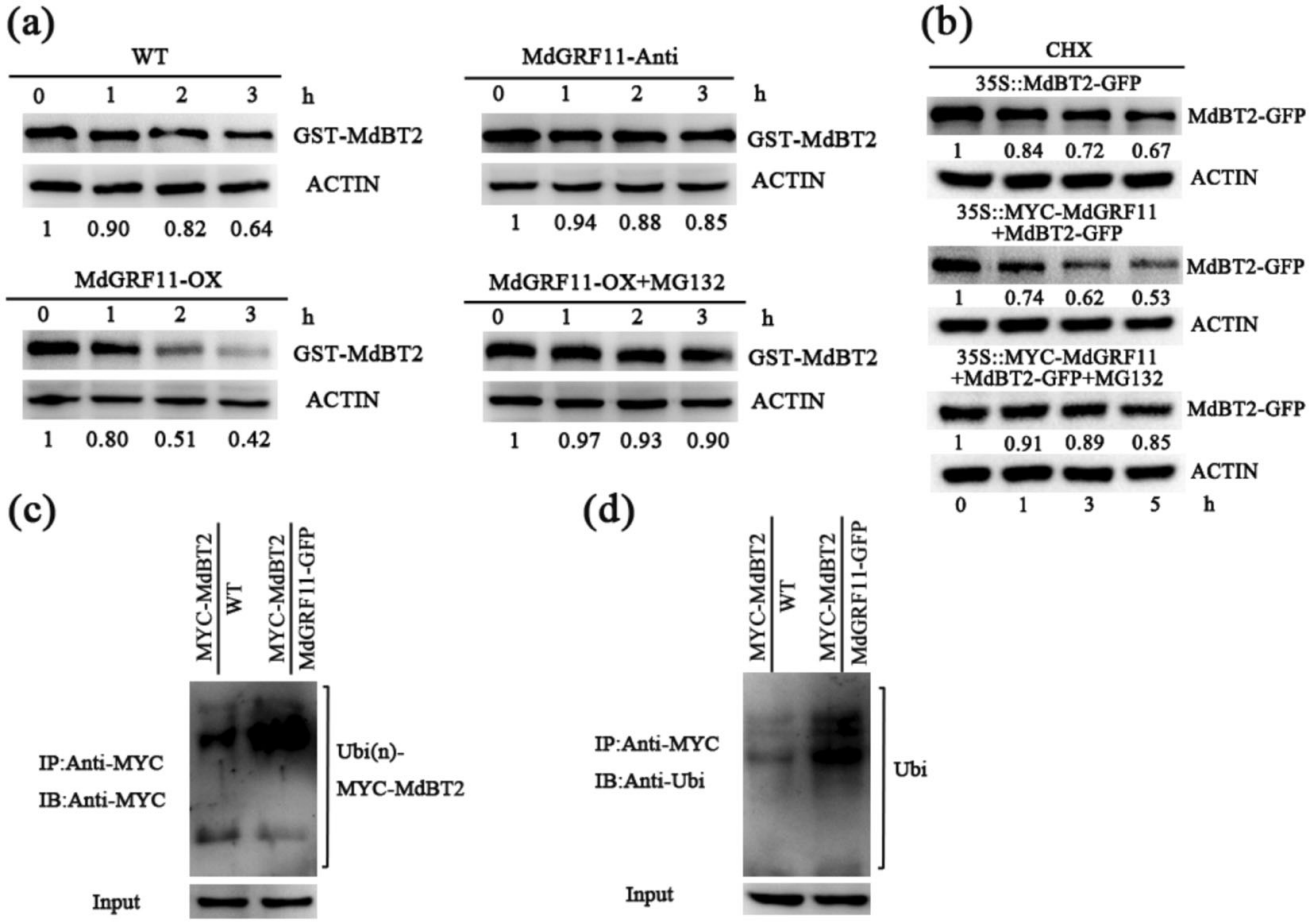
MdGRF11 promotes the destabilization of MdBT2. **a** In vitro cell-free degradation assay in WT control, *MdGRF11-OX*, and *MdGRF11-Anti* transgenic apple calli. Equal quantities of recombinant GST-MdBT2 protein and total protein samples extracted from WT, *MdGRF11-Anti*, or *MdGRF11-OX* apple calli treated with or without MG132 were incubated together at 22 °C for the indicated period. Anti-GST antibody was used to detect GST-MdBT2 protein abundance. **b** In vivo MdBT2 protein degradation assay. The MdBT2-GFP protein abundances in two-week-old *MdBT2-GFP* and *MYC-MdGRF11* + *MdBT2-GFP* transgenic calli were detected with an anti-GFP antibody according to the indicated time after treatment with 250 μM cycloheximide (CHX) with or without MG132 in the dark. ACTIN was used as a loading control. The protein levels at 0 h were set to 1. **c**, **d** Ubiquitination of MdBT2 protein in *MYC-MdBT2* and *MdGRF11-GFP* + *MYC-MdBT2* transgenic calli. MYC-MdBT2 proteins were immunoprecipitated using the anti-MYC antibody from these two transgenic calli after pretreatment with 50 μM MG132 in the dark for 6 h. The immunoprecipitated proteins were detected with anti-MYC antibodies (**c**) and anti-ubiquitin antibodies (**d**). IB immunoblotted, IP immunoprecipitated

In animals, 14-3-3 proteins have been reported to mediate the stability of their target proteins by direct protein–protein interactions^51^. To investigate the influence of the interaction between MdGRF11 and MdBT2 on the stability of MdBT2, a cell-free degradation experiment was conducted. Purified GST-MdBT2 proteins were added to total protein samples extracted from WT control, *MdGRF11-OX*, and *MdGRF11-Anti* transgenic calli. Western blot analysis demonstrated that GST-MdBT2 proteins degraded faster in *MdGRF11-OX* protein extracts than in the WT control extracts but much slower in *MdGRF11-Anti* protein extracts (Fig. 5a), suggesting that MdGRF11 contributed to the degradation of MdBT2 proteins. Furthermore, MdGRF11-mediated degradation of MdBT2 was noticeably inhibited in the presence of MG132 (Fig. 5a). Taken together, these results revealed that the degradation of MdBT2 was dependent on the 26S proteasome pathway.

To further verify the influence of MdGRF11 on MdBT2 proteins in vivo, *MYC-MdGRF11* was transformed into *MdBT2-GFP* transgenic calli to generate *MYC-MdGRF11* + *MdBT2-GFP* double transgenic calli (Supplementary Fig. 5a). The *MdBT2-GFP* and *MYC-MdGRF11* + *MdBT2-GFP* calli were then treated with the protein synthesis inhibitor cycloheximide (CHX) for different durations to examine the abundance of MdBT2. Western blotting assays showed that the degradation of MdBT2 was accelerated in the *MYC-MdGRF11* + *MdBT2-GFP* calli compared with the *MdBT2-GFP* calli. However, MG132 notably inhibited the degradation of MdBT2 in the *MYC-MdGRF11* + *MdBT2-GFP* calli (Fig. 5b), indicating that MdGRF11 negatively regulated MdBT2 protein stability via the 26S proteasome-dependent pathway.

To determine whether MdGRF11-mediated degradation of MdBT2 depended on the ubiquitin/proteasome pathway, MdBT2 proteins were immunoprecipitated from *MYC-MdBT2* and *MdGRF11-GFP* + *MYC-MdBT2* calli with anti-MYC antibodies. The results indicated that *MdGRF11-GFP* + *MYC-MdBT2* calli generated more of the polyubiquitinated form MYC-MdBT2 than did the *MYC-MdBT2* calli (Fig. 5c, d). Thus, MdGRF11 promoted the ubiquitination and subsequent degradation of the MdBT2 protein.

Generally, BTB proteins degrade their target proteins by combining with CULLIN3 to form the CULLIN-3 E3 (CUL3) ubiquitin ligase complex^52^. To confirm whether MdBT2 affected the protein stability of MdGRF11, protein degradation assays were conducted in vitro. The suppression vector *MdBT2-Anti* and the overexpression vector *MdBT2-OX* were introduced into the apple calli (Supplementary Fig. 5b). The cell-free assays showed that there was no significant difference in the degradation rate of HIS-MdGRF11 among the WT control, *MdBT2-OX*, and *MdBT2-Anti* callus protein extracts (Supplementary Fig. 6). These data indicated that MdBT2 did not affect the stability of the MdGRF11 protein, indicating that the MdGRF11 14-3-3 protein was not a substrate of MdBT2.

### MdGRF11 negatively regulates MdBT2-mediated anthocyanin accumulation

**Fig. 6:**
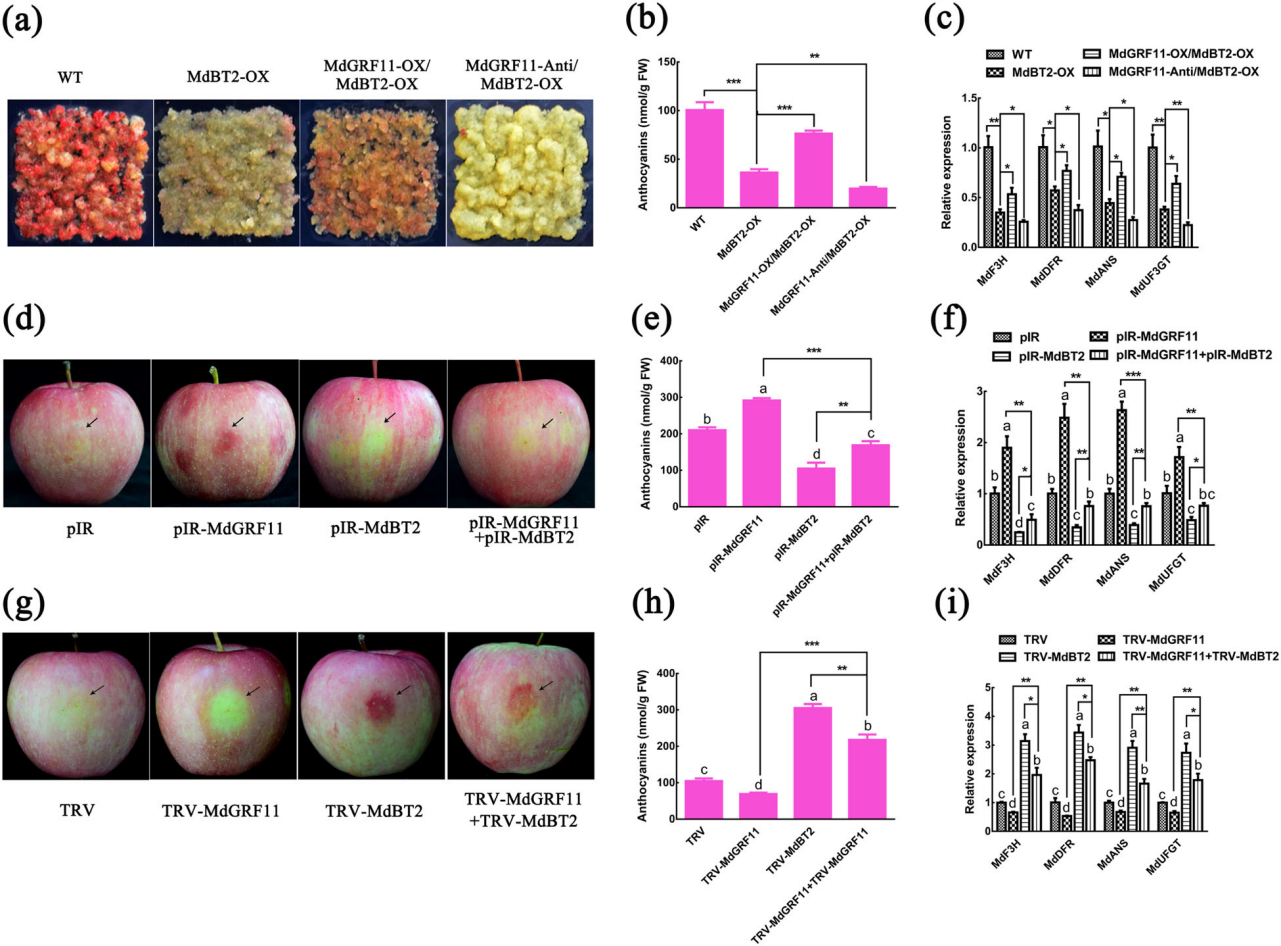
MdGRF11 negatively regulates MdBT2-mediated anthocyanin accumulation. **a** Coloration of WT, *MdBT2-OX*, *MdGRF11-OX* + *MdBT2-OX*, and *MdGRF11-Anti* + *MdBT2-OX* transgenic calli treated with 5 mM KCl for 7 days. **b** Anthocyanin contents in the corresponding calli shown in **a**. **c** Relative expression levels of the anthocyanin biosynthesis-related genes *MdF3H*, *MdDFR*, *MdANS*, and *MdUF3GT* in the apple calli shown in **a**. The *18s* gene acted as an internal control. **d** Coloration of apple fruit peels injected with plasmid mixtures (pIR-MdGRF11, pIR-MdBT2, and pIR-MdGRF11+pIR-MdBT2). An empty pIR vector was used as control. **e, f** Anthocyanin contents (**e**) and relative expression levels of the anthocyanin biosynthesis-related genes *MdF3H*, *MdDFR*, *MdANS*, and *MdUF3GT* (**f**) around the injection sites of the fruit peels shown in **d**. The *18s* gene acted as an internal control. **g** Coloration of apple fruit peels injected with a mixed solution of *Agrobacterium* cells (TRV-MdGRF11, TRV-MdBT2, and TRV-MdGRF11+TRV-MdBT2). An empty TRV vector was used as a control. **h**, **i** Anthocyanin contents (**h**) and transcript levels of anthocyanin biosynthesis-related genes (**i**) around the injection sites of the fruit peels shown in **g**. The *18s* gene acted as an internal control. In **b**, **c**, **e**, **f**, **h**, **i**, the error bars indicate the SDs of three independent experiments, each of which included three technical replicates. The different letters represent significant differences (LSD test, *P* < 0.05). The asterisks indicate significant differences (*P < 0.5; **P < 0.01; ***P < 0.001) based on Student’s *t* test

Considering that both MdGRF11 and MdBT2 are involved in nitrate deficiency-mediated anthocyanin accumulation and that MdGRF11 contributes to the degradation of MdBT2, it was reasonable to suspect that MdGRF11 influences MdBT2-mediated anthocyanin biosynthesis. Thus, *MdGRF11-Anti* and *MdGRF11-OX* constructs were introduced into *MdBT2-OX* calli (Supplementary Fig. 5c). The resultant *MdBT2-OX*, *MdGRF11-OX* + *MdBT2-OX*, and *MdGRF11-Anti* + *MdBT2-OX* transgenic calli were used to detect the anthocyanin accumulation in KCl-treated media, while WT calli were used as a control. Overexpression of *MdBT2* noticeably decreased the anthocyanin accumulation. Additionally, overexpression of *MdGRF11* partially suppressed, whereas suppression of *MdGRF11* promoted MdBT2-mediated anthocyanin accumulation (Fig. 6a, b). Expression analysis showed that the expression levels of the anthocyanin biosynthesis-related genes *MdF3H*, *MdDFR*, *MdANS*, and *MdUF3GT* were markedly downregulated in *MdBT2-OX* calli, and this phenomenon could be altered by *MdGRF11* overexpression or suppression (Fig. 6c). Taken together, these results demonstrated that MdGRF11 negatively regulated MdBT2-mediated anthocyanin biosynthesis in apple calli.

Previous studies have shown that MdBT2 inhibits anthocyanin accumulation by promoting MdMYB1 degradation^25^. Therefore, it was also hypothesized that MdGRF11 regulated MdBT2-mediated MdMYB1 protein stability to affect anthocyanin biosynthesis. The cell-free degradation assay showed that the degradation rate of HIS-MdMYB1 proteins was faster in protein extracts of *MdBT2-OX* than in those the WT control. When MdGRF11 was suppressed in the background of *MdBT2-OX* calli, the degradation of HIS-MdMYB1 proteins was accelerated in the *MdGRF11-Anti* + *MdBT2-OX* protein extracts. However, overexpression of *MdGRF11* nearly prevented MdBT2-induced HIS-MdMYB1 degradation in the *MdGRF11-OX* + *MdBT2-OX* protein extracts (Supplementary Fig. 7), suggesting that MdGRF11-regulated anthocyanin biosynthesis was at least partly dependent on the MdBT2-MdMYB1 regulatory module.

To explore the role of MdBT2 and MdGRF11 in apple peel coloration, viral vector-mediated transient injection assays were carried out in apple skins. qRT-PCR analysis showed that the transcript levels of *MdBT2* and *MdGRF11* correspondingly increased or decreased in response to injection of apple skins (Supplementary Fig. 8). As a result, overexpression of *MdBT2* (pIR-MdBT2) inhibited anthocyanin biosynthesis in apple skins around the infiltration sites, while overexpression of *MdGRF11* (pIR-MdGRF11) promoted the biosynthesis compared with that of the controls. In addition, when pIR-MdGRF11 and pIR-MdBT2 were coinjected together, the anthocyanin accumulation was higher than that of pIR-MdBT2 alone but still lower than that of the pIR control (Fig. 6d, e). Coinjection of TRV-MdGRF11 and TRV-MdBT2 resulted in increased anthocyanin accumulation compared with that resulting from the TRV control, as did TRV-MdBT2 (Fig. 6g, h). Moreover, the trends of the relative expression levels of the structural genes *MdF3H*, *MdDFR*, *MdANS*, and *MdUF3GT* were similar to the trends of anthocyanin accumulation in the injected fruit peels (Fig. 6f, i).

The overexpression viral vector pIR-MdGRF11 and suppression viral vector TRV-MdGRF11 were subsequently used for transient infection of GL-3 apple leaves. The results indicated that MdGRF11 promoted anthocyanin accumulation in apple leaves (Supplementary Fig. 9a–c). Moreover, pIR-MdGRF11 and TRV-MdGRF11 were transiently infected into apple leaves of *MdBT2* overexpression transgenic apple lines (*MdBT2-OX1*, *OX5*, and *OX7*) and suppression lines (*MdBT2-Anti13* and *Anti23*), with pIR and TRV empty vectors serving as controls. These results indicated that MdGRF11 inhibited MdBT2-mediated anthocyanin biosynthesis in apple leaves (Supplementary Fig. 9d–k), similar to the findings pertaining to the apple calli and apple fruits.

## Discussion

Anthocyanin biosynthesis is regulated by various signal transduction pathways that integrate multiple hormone and environmental signals, such as nitrate^9,15,16^. Nitrate deficiency-induced anthocyanin biosynthesis has been reported in many species, including *Arabidopsis* and apple, and many proteins have been identified to participate in this process, including MYB, bHLH, LBD, and NLA proteins^17,20,21,23^. In apple, the nitrate-responsive protein MdBT2 degrades MdMYB1 proteins, which inhibit MdMYB1-induced anthocyanin accumulation under adequate nitrogen conditions^25^. However, the mechanisms underlying MdBT2-MdMYB1 pathway suppression under nitrate-deficiency conditions remain elusive. In this study, we found that the 14-3-3 protein MdGRF11 promotes the degradation of the MdBT2 protein, thereby enhancing the abundance of the MdMYB1 protein, which in turn induces anthocyanin accumulation in response to nitrate deficiency.

As a substrate adapter of Cullin-RING E3 ubiquitin ligase (CRL3), MdBT2 can modulate the stability of multiple proteins in response to environmental signals^30,52^. Recent studies have demonstrated that MdBT2 negatively regulates anthocyanin biosynthesis by modulating the abundance of the MdMYB1, MdBBX22, MdWRKY40, MdERF38, and MdTCP46 proteins in response to nitrate, UV-B, wounding, drought stress, and high light, respectively^25,36–39^. MdBT2 also undergoes ubiquitination in response to external environmental factors^36–39^ (Fig. 1d). In mammalian cells, some E3 ligase components are ubiquitinated and degraded through the 26S proteasome. For example, the F-box protein Atrogin-1 is ubiquitinated and degraded through the p38 mitogen-activated protein kinase-dependent pathway^53^. Likewise, the anaphase-promoting complex promotes the degradation of the F-box protein S-phase kinase-associated protein 2 (Skp2) in the cell cycle phase G1^54^. In *Arabidopsis*, interactions with 14-3-3 proteins may enhance the self-ubiquitination activity of the F-box protein FBS1^55^. Here, the 14-3-3 protein MdGRF11 was shown to interact with and promote the destabilization of the BTB protein MdBT2 (Figs. 2, 5). The number of identified gene families encoding E3 ligase components in plants has greatly increased, and the number of their related functions may increase. The regulation of BT2 protein stability may imply the function of scaffold proteins in bridging the gap between the response to environmental signals and the regulation of downstream target proteins, representing an increase in the complexity of the plant 26S proteasome system.

14-3-3 proteins are highly conserved phosphopeptide-binding proteins that participate in multiple physiological and biological processes, including the stress response, primary metabolism, signal transduction, and membrane transport^56,57^. Recent studies have indicated that the 14-3-3 lambda (14-3-3λ) protein regulates anthocyanin biosynthesis during drought stress^58^. In apple, a total of 20 14-3-3 genes have been identified to encode functional proteins^41^. Among them, the expression pattern of MdGRF11 was opposite that of MdBT2 in response to nitrate deficiency (Fig. 3b–d). Overexpression of *MdGRF11* promoted nitrate deficiency-induced anthocyanin biosynthesis in transgenic calli and *Arabidopsis* (Fig. 4). Anthocyanin biosynthesis plays a vital role in the antioxidant defense mechanism to scavenge excess reactive oxygen species (ROS)^59^. Therefore, anthocyanin accumulation in *MdGRF11* overexpression transgenic lines may explain the role of MdGRF11 in positively regulating stress tolerance to some extent^41^. 14-3-3 proteins have high functional redundancy, which is a primary obstacle to studying the function of individual 14-3-3 isoforms^57^. Consistent with this, MdBT2 was found to interact with multiple 14-3-3 isoforms (Supplementary Fig. 2a), and the *grf9* mutant did not exhibit a significant difference in anthocyanin content compared with that of the control (Col-0) (Fig. 4g, h). However, *MdGRF11-Anti* transgenic calli, which repressed the expression of multiple 14-3-3 isoforms, accumulated markedly lower anthocyanin levels than did the WT control (Fig. 4a, b), suggesting that the redundancy of 14-3-3 proteins was at play in anthocyanin biosynthesis.

Generally, 14-3-3 proteins interact with target proteins and mediate their characteristics, such as protein activity, stability, subcellular localization, conformation, and affinity for other proteins^50,51,57^. In mammalian cells, 14-3-3 proteins promote the self-ubiquitination and degradation of the E3 ligases MURINE DOUBLE MINUTE2 (MDM2) and CONSTITUTIVE PHOTOMORPHOGENIC 1 (COP1), thus enhancing the stability of p53, a short-lived tumor suppressor protein^60,61^. In *Arabidopsis*, 14-3-3 proteins have also been shown to positively regulate the stability of the type-2 1-aminocyclopropane-1-carboxylate synthase (ACS) protein. The regulation is partially caused by decreasing the abundance of BTB proteins ETHYLENE-OVERPRODUCER 1 (ETO1)/ETO1-LIKE (EOLs), which target type-2 ACS proteins for ubiquitination and degradation^62^. Here, we describe a similar mechanism in which the 14-3-3 protein MdGRF11 negatively regulated BTB protein MdBT2 stability in a 26S proteasome-dependent pathway and thus increased the abundance of MdMYB1 proteins to promote anthocyanin biosynthesis under nitrate-deficiency conditions (Figs. 5, 6 and Supplementary Fig. 7).

Previous studies have demonstrated that the yeast F-box proteins Met30p, Grr1p, and Cdc4p are unstable and undergo ubiquitination and degradation via an autocatalytic mechanism within their own SCF complex, thereby ensuring that cells quickly adapt to extracellular signals and the progression of the cell cycle^63^. Furthermore, F-box proteins dimerize within the assembled SCF complex, which contributes to the stable binding of the F-box protein to its substrate and to its effective ubiquitination^64,65^. The BTB proteins MEL-26 and Keap1 also dimerize, and this dimerization makes them subject to degradation^66,67^. 14-3-3 proteins mainly exist in a homo-/heterodimeric form, and they binds to target proteins through a phosphorylated serine/threonine, thereby exerting their regulatory function^50,57^. It was demonstrated that 14-3-3 proteins promote F-box protein Fbx4 dimerization following Fbx4 phosphorylation, thereby enhancing the E3 ligase activity of SCF^Fbx4^^68^. It is thus possible that interaction with dimeric 14-3-3 proteins may promote the dimerization of CRL3, hence stimulating MdBT2 self-ubiquitination and degradation via the 26S proteasome pathway. Moreover, phosphorylation of MdBT2 could generate a 14-3-3 binding site, though this has not been demonstrated in MdBT2 interactions. Furthermore, identification of the phosphorylation site of MdBT2 and the potential kinases involved would provide new insights into the mechanism underlying MdBT2 protein modifications in response to nitrate deficiency.

Previous studies have demonstrated that 14-3-3 proteins are central components in the plant signaling network for integrating various external and internal inputs^56^. 14-3-3 s mediate the stability of target proteins through direct interactions, and this action may be a universal mechanism for regulating signaling components in plants. Additionally, the regulation of E3 ligase component stability may play a vital role in plant signaling regulatory networks.

## Supplementary information

Supplementary Information
