# Supplementary material for: The apple 14-3-3 protein MdGRF11 interacts with the BTB protein MdBT2 to regulate nitrate deficiency-induced anthocyanin accumulation
Source: Hortic Res. 2021 Feb 1;8:22. doi: 10.1038/s41438-020-00457-z (PMC7848006; doi:10.1038/s41438-020-00457-z)
Supplement: Supplementary file 1 — Supplementary Information [file 41438_2020_457_MOESM1_ESM.doc]

**Supplementary Information**

**Supplementary Fig. 1 MdBT2 regulates anthocyanin accumulation. a** Anthocyanin pigmentation phenotype of 4-week-old wild-type (WT), *MdBT2* overexpression (*MdBT2-OX1, OX5* and *OX7*) and suppression expression (*MdBT2-Anti13* and *Anti23*) transgenic apple tissue culture seedlings treated with 5 mM KCl under constant light for 10 d. **b, c** Phenotypes (b) and anthocyanin contents (c) of apple leaves in apple tissue culture seedlings shown in (a). **d-f** Expression analysis of anthocyanin biosynthesis genes *MdDFR*, *MdANS* and *MdUF3G* with qRT-PCR in apple tissue culture seedlings shown in (a). *18S* acted as the internal control. In (c-f), error bars indicate SD based on three independent experiments with at least 6 plants for each experiment. Asterisk indicates significant differences (**P* < 0.5, ***P* < 0.01, ****P* < 0.001, based on Student’s *t*-test).


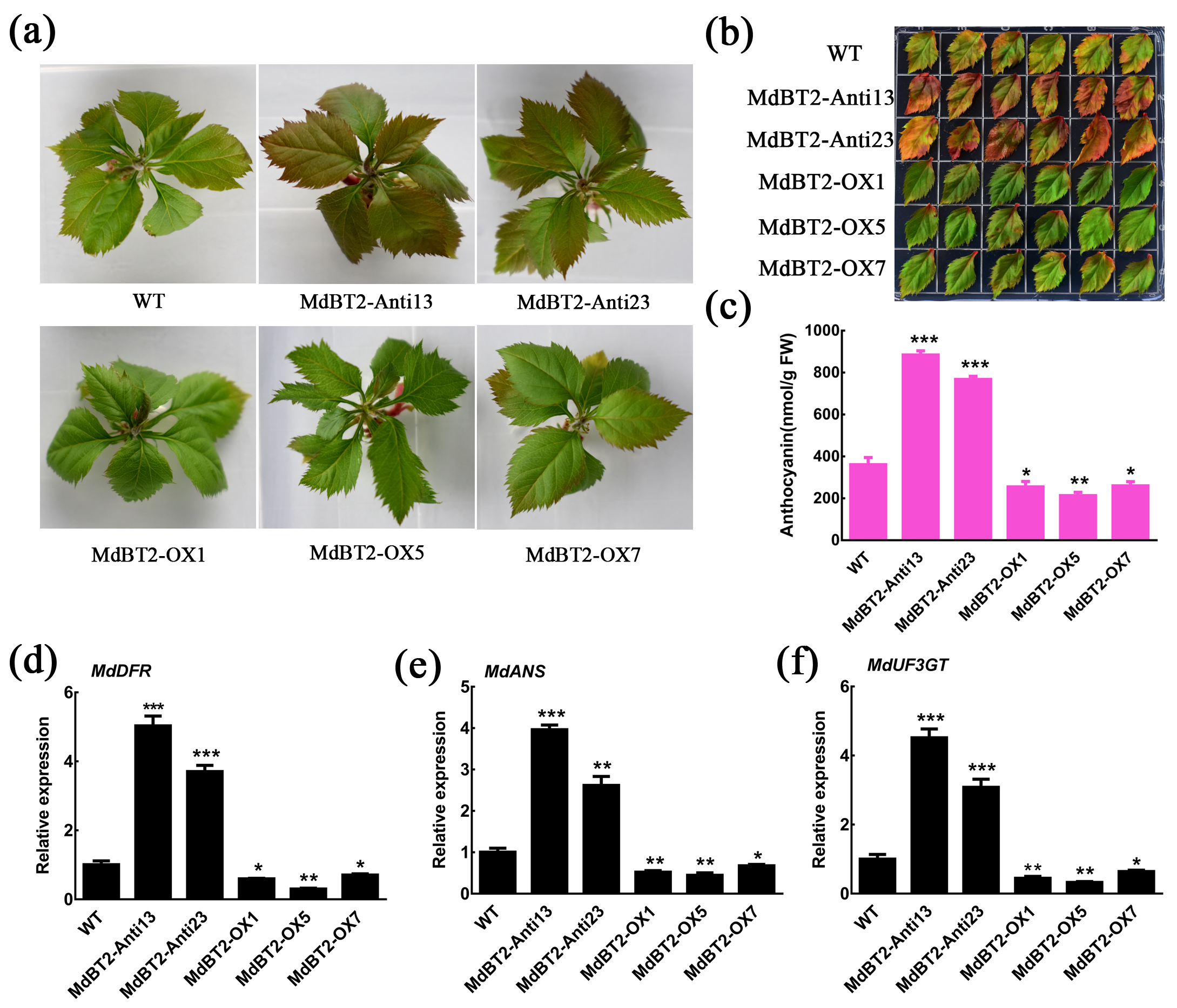


**Supplementary Fig. 2 The interaction between BTB-TAZ and GRF proteins. a** Y2H assay to examine interactions of MdBT2 with the MdGRF4, MdGRF6 and MdGRF18. **b** Y2H assay for the interaction between MdGRF11 and AtBTs. **c** Interactions of AtBT2 with AtGRFs in yeast cells.


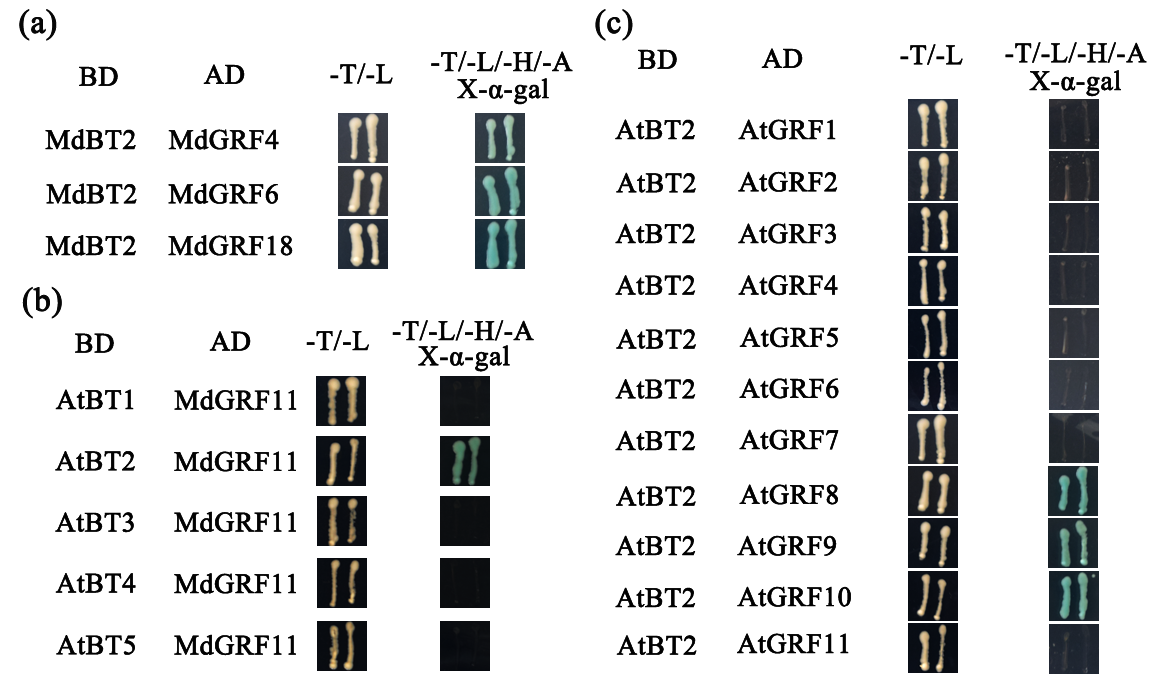


**Supplementary Fig. 3 Identification of *MdGRF11-Anti* transgenic apple calli and *grf9* mutant *Arabidopsis*. a** qRT-PCR analysis of *MdGRF4*, *MdGRF6* and *MdGRF18* expression in ‘Orin’ apple calli (WT) and *MdGRF11-Anti* transgenic calli. **b** The transcripts of *AtGRF9* in Col-0 and *grf9* mutant. *18S* was used as a control, and error bars indicate SD based on three independent biological replicates.

**
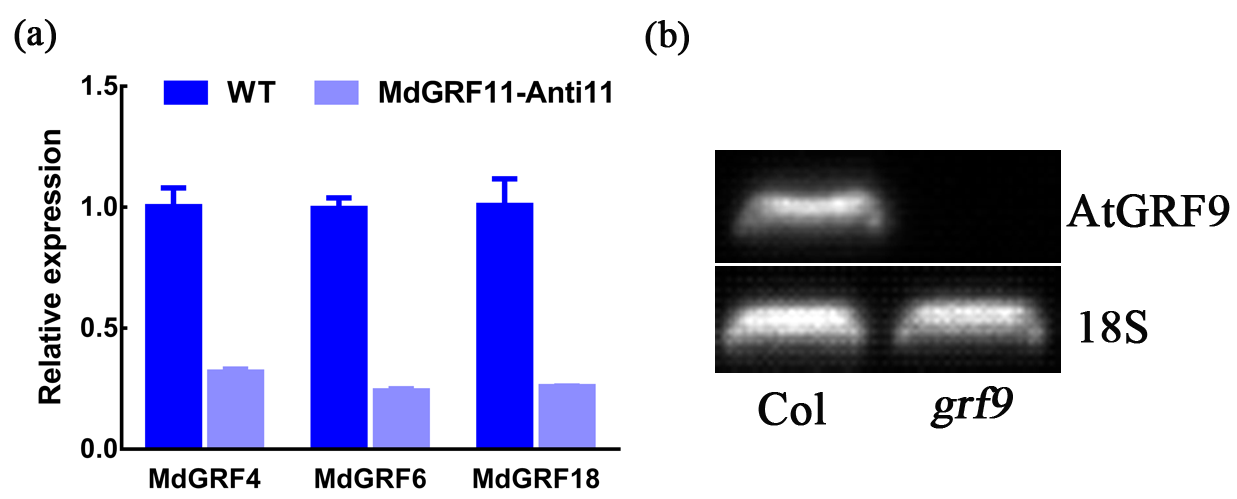
**

**Supplementary Fig. 4 Nitrate inhibits anthocyanin biosynthesis in *Arabidopsis.* a** Coloration of *Arabidopsis* seedlings treated with different concentrations of nitrate as indicated for 7 d. **b** Root lengths of *Arabidopsis* seedlings in (a). **c** Anthocyanin contents of *Arabidopsis* seedlings in (a). In (b, c), error bars represent SD based on three independent experiments, and each of which has three technical replicates. Different letters represent significant differences (LSD test, *P* < 0.05).


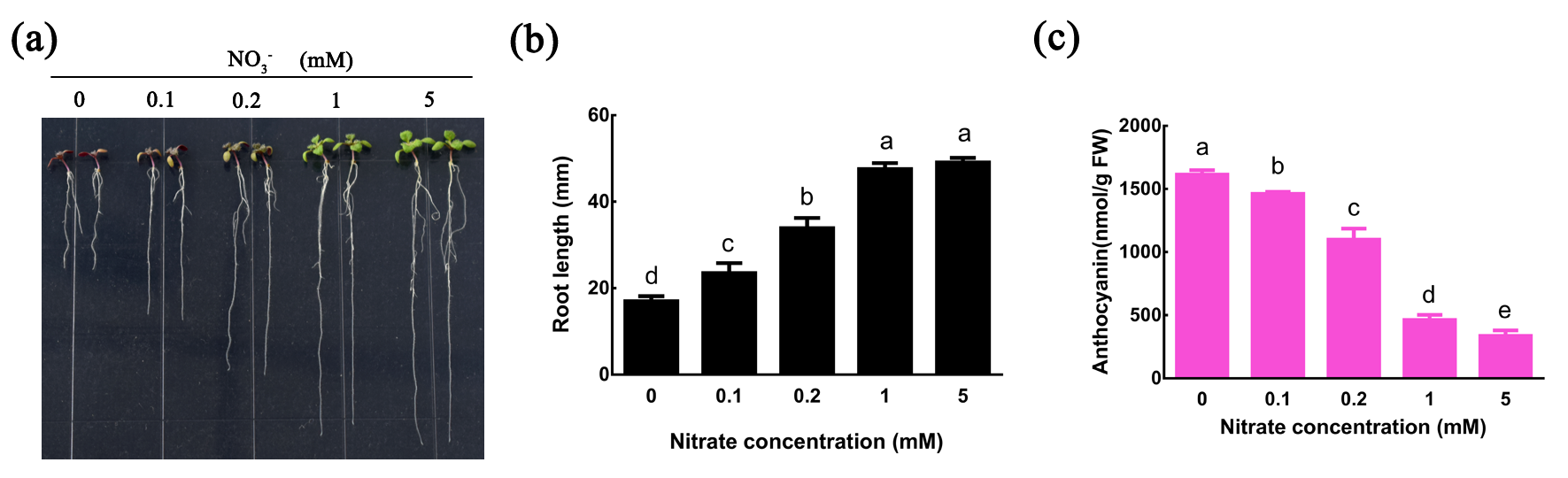


**Supplementary Fig. 5 Expression analysis of *MdBT2* and *MdGRF11* in different transgenic apple calli. a** qRT-PCR analysis of *MdGRF11* expression in *MdBT2-GFP* and *MYC-MdGRF11+MdBT2-GFP* transgenic calli. **b** qRT-PCR analysis of *MdBT2* expression in ‘Orin’ apple calli (WT), *MdBT2-OX* and *MdBT2-Anti* transgenic calli. **c** Transcript levels of *MdGRF11* in *MdBT2-OX*, *MdGRF11-OX*+*MdBT2-OX*, and *MdGRF11-Anti*+*MdBT2-OX* transgenic calli with qRT-PCR. *18S* was used as internal control, and error bars indicate SD based on three independent biological replicates.

**
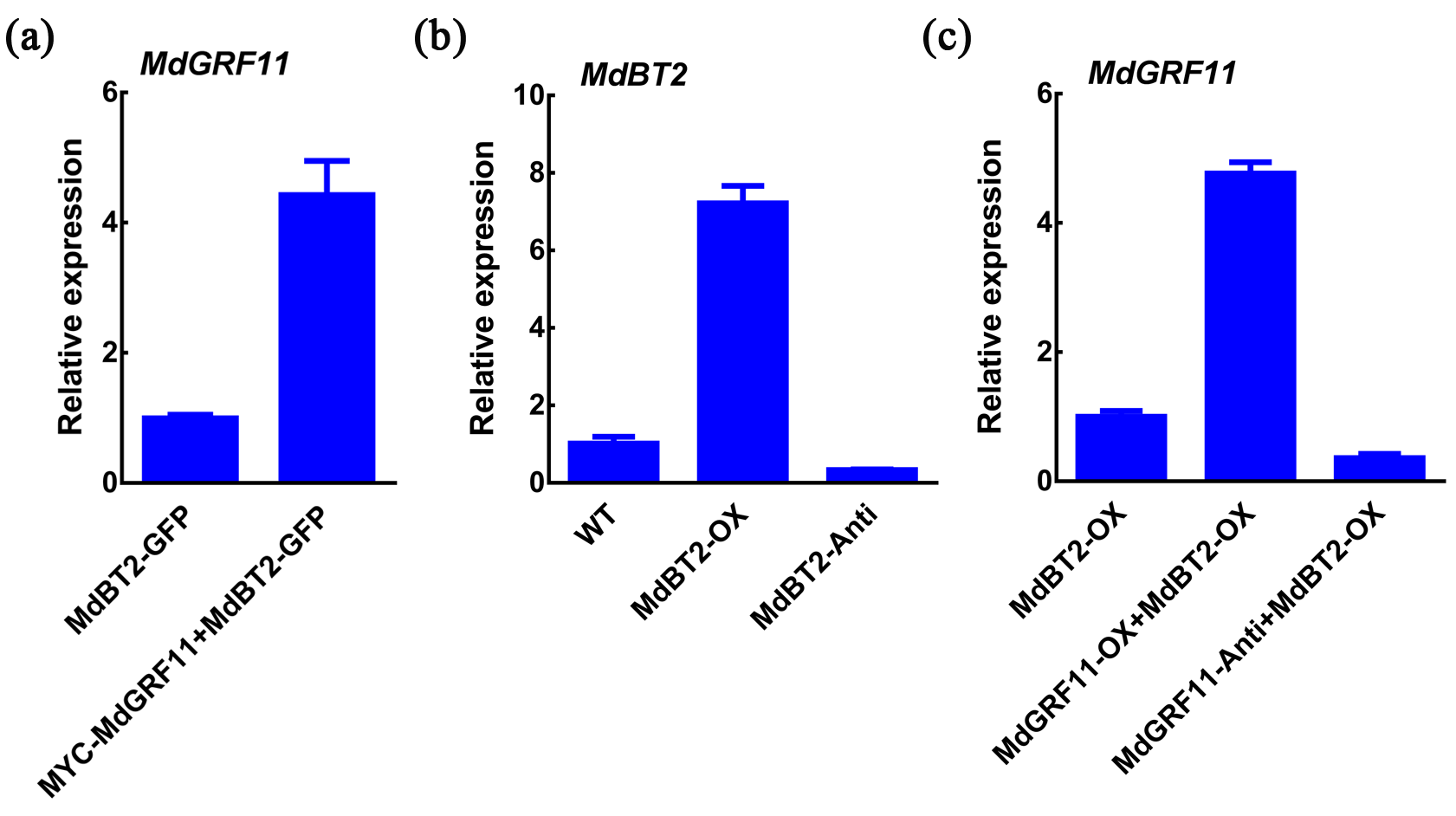
**

**Supplementary Fig. 6 MdBT2 has no effect on MdGRF11 protein stability.** Cell-free degradation assay of the recombinant HIS-MdGRF11 protein in the protein extracts of apple calli as labeled. Total proteins extracts from WT control, *MdBT2-OX* and *MdBT2-Anti* calli were incubated with purified HIS-MdGRF11 protein at 22 °C. The samples were harvested at the indicated time, and then analyzed by immunoblotting using an anti-GST antibody. ACTIN was used as a loading control. The protein levels at 0 h were set to 1.


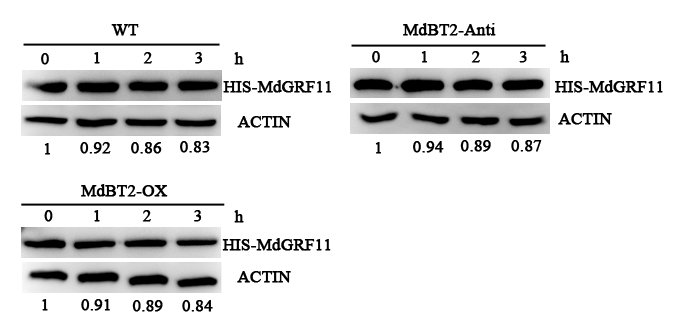


**Supplementary Fig. 7 MdGRF11 regulates MdBT2-mediated MdMYB1 protein stability.** *In vitro* cell-free degradation assay in WT control, *MdBT2-OX*, *MdGRF11-Anti*+*MdBT2-OX* and *MdGRF11-OX*+*MdBT2-OX* transgenic apple calli. HIS-MdMYB1 fusion proteins were incubated with equal amounts of total protein extracts of apple calli as labeled for the indicated times. The levels of HIS-MdMYB1 were detected by immunoblotting using an anti-HIS antibody. ACTIN was used as an internal reference. The protein levels at 0 h were set to 1.


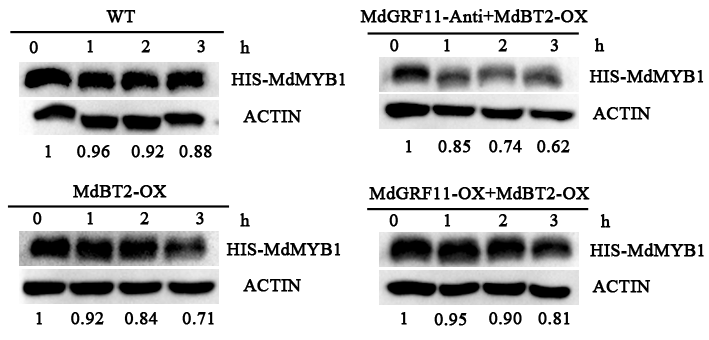


**Supplementary Fig. 8 Expression analysis of *MdGRF11* and *MdBT2* in fruit peels around the injection sites. a, b** qRT-PCR analyzes the transcript levels of *MdGRF11* (a) and *MdBT2* (b) in apple skins injected with mixed plasmids (pIR, pIR-MdGRF11, pIR-MdBT2 and pIR-MdGRF11+pIR-MdBT2). **c, d** qRT-PCR analyzes the transcript levels of *MdGRF11* (c) and *MdBT2* (d) in apple skins injected with mixed *Agrobacterium* solutions (TRV, TRV-MdGRF11, TRV-MdBT2 and TRV-MdGRF11+TRV-MdBT2). *18S* acted as the internal control. The error bars indicate SD based on three independent experiments, and each of which has three technical replicates. Asterisk indicates significant differences (**P* < 0.5, ***P* < 0.01, based on Student’s *t*-test).


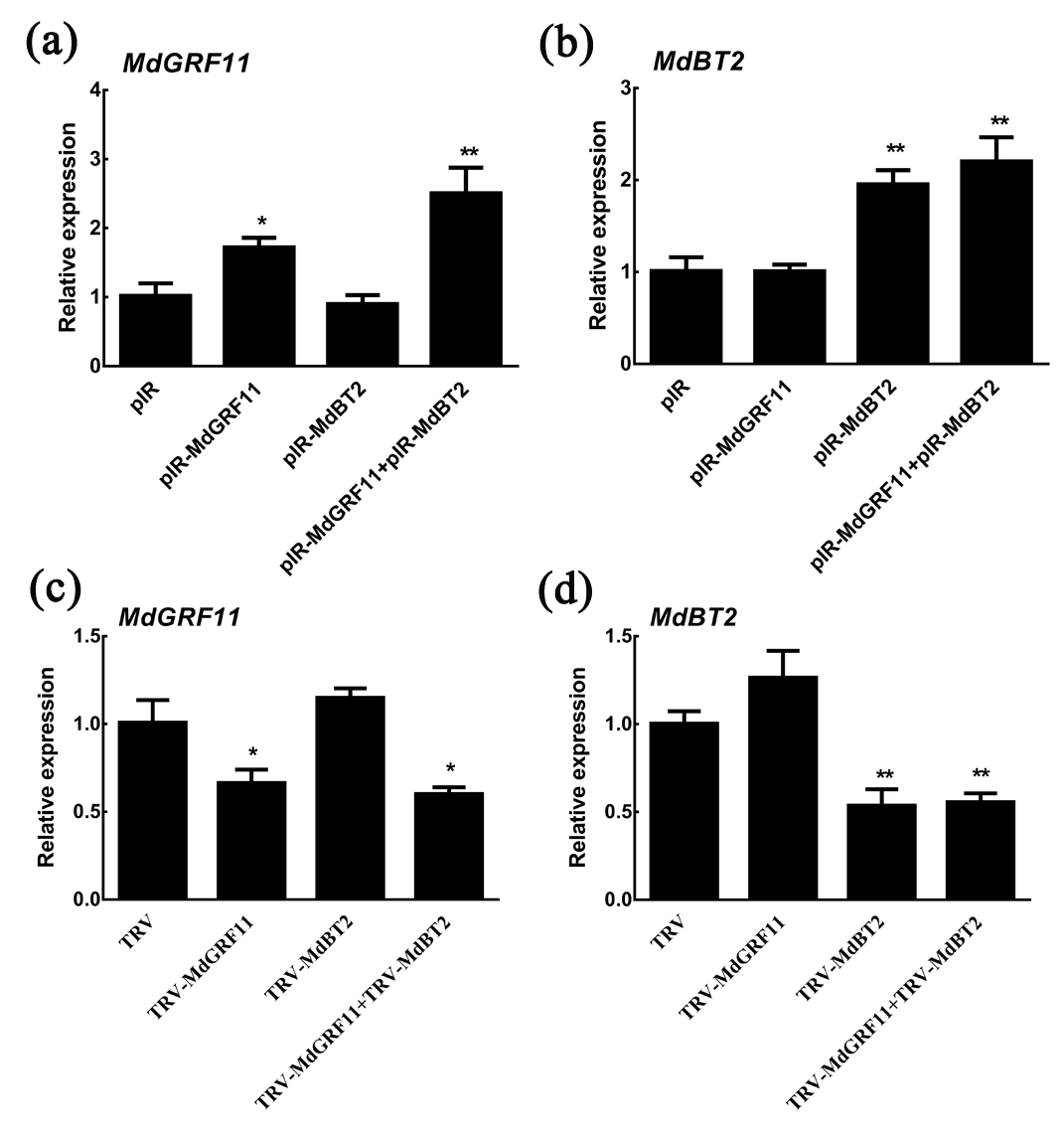


**Supplementary Fig. 9 MdGRF11 negatively regulates MdBT2-mediated anthocyanin accumulation. a** Phenotypes of GL-3 apple leaves infected by viral vectors pIR-MdGRF11 and TRV-MdGRF11 in KCl-treated medium under constant light for 5 d. The empty vectors pIR and TRV were used as controls. **b, c** The expression level of *MdGRF11* (b) and anthocyanin content (c) in the apple leaves shown in (a). **d, e** Phenotypes of *MdBT2* suppression expression transgenic apple leaves (*MdBT2-Anti13* and *Anti23*) infected by viral vectors pIR-MdGRF11 (d) and TRV-MdGRF11 (e). The empty vectors pIR and TRV were used as controls. **f, g** The expression level of *MdGRF11* (f) and anthocyanin content (g) in the apple leaves shown in (d and e). **h, i** Phenotypes of *MdBT2* overexpression transgenic apple leaves (*MdBT2-OX1, OX5* and *OX7*) infected by viral vectors pIR-MdGRF11 (h) and TRV-MdGRF11 (i). The empty vectors pIR and TRV were used as controls. **j, k** The expression level of *MdGRF11* (j) and anthocyanin content (k) in the apple leaves shown in (h and i). *18S* acted as the internal control. In (b, c, f, g, j, k), error bars indicate SD based on three independent experiments, and each of which has three technical replicates. Asterisk indicates significant differences (**P* < 0.5, ***P* < 0.01, based on Student’s *t*-test).


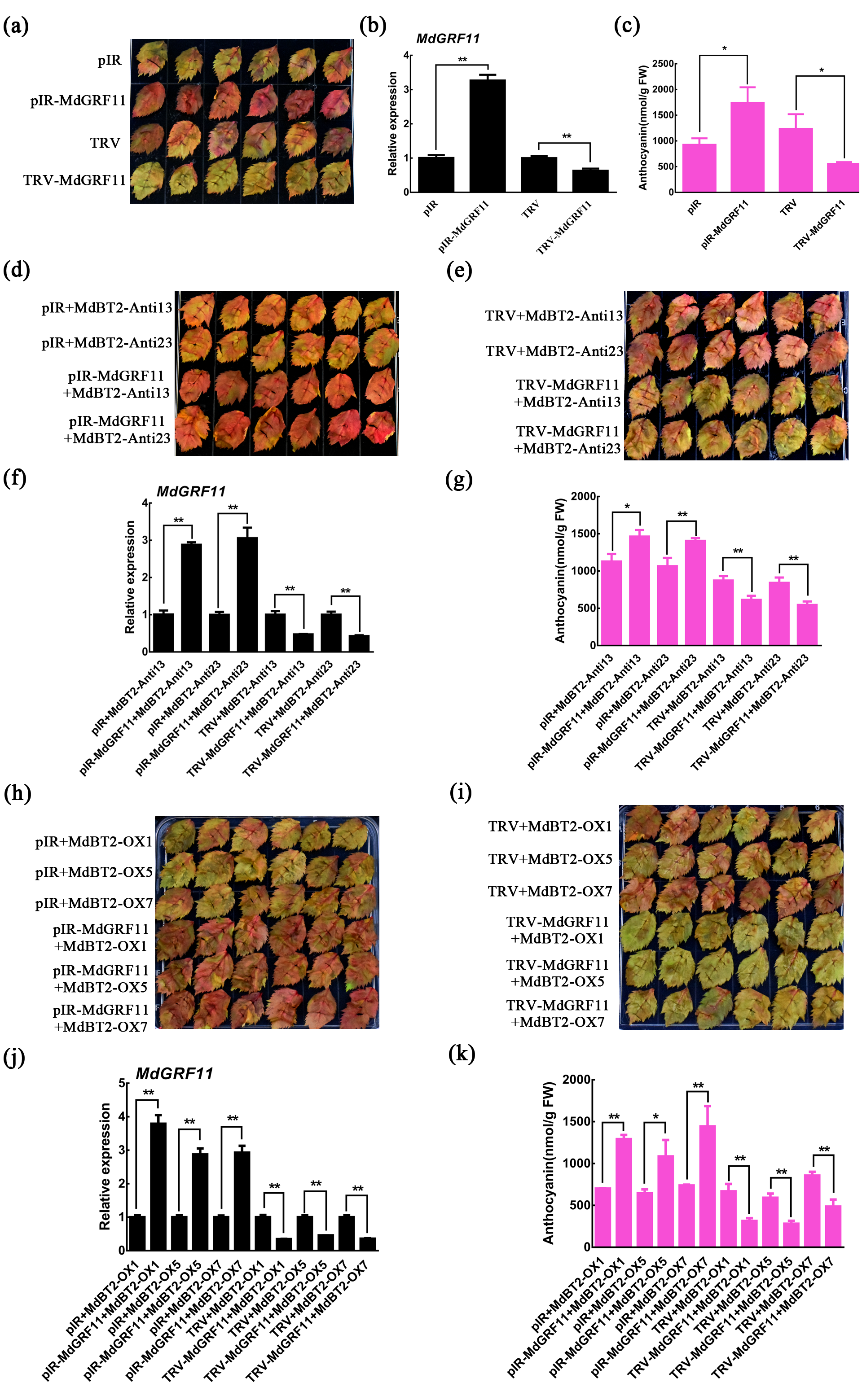


**Supplementary Table 1 Primers used in this study.**

| **Primer names for genes** | **Primer sequence (5'→3')** |
| --- | --- |
| MdGRF4 (AD)-5 | GGATCCAAATGGCTTCCCCCA |
| MdGRF4 (AD)-3 | GTCGACTCACTCTGCATCTTCA |
| MdGRF6 (AD)-5 | CCCGGGAATGTCGCCAACTGAT |
| MdGRF6 (AD)-3 | GTCGACTTACTGCTGTGCTTCA |
| MdGRF11 (AD)-5 | CCCGGGAATGGGGCCGTACGGTTCC |
| MdGRF11 (AD)-3 | GTCGACCTCTGCATCTTCACCTCCA |
| MdGRF18 (AD)-5 | CCCGGGAATGTCGCCACCTGAT |
| MdGRF18 (AD)-3 | GTCGACTTACTGTGCTTCACTGGA |
| MdBT1 (BD)-5 | CGTCGACAGTTATACTTGCTCCTGG |
| MdBT1 (BD)-3 | CAGATCTCACGTCCACAATGAACATG |
| MdBT2 (BD)-5 | CCCGGGAATGGAAGCTAATCCGACC |
| MdBT2 (BD)-3 | CTGCAGCAATCTGAAGCTTCTAAT |
| MdBT3.1 (BD)-5 | ATGGCTTCATCTACTCCGG |
| MdBT3.1 (BD)-3 | TCATGATAAACGCGAGTGG |
| MdBT3.2 (BD)-5 | ATGGCTTCACCTACTCTTG |
| MdBT3.2 (BD)-3 | TCATGAAAAACGTGAGCGC |
| MdBT4 (BD)-5 | ATGTGTAAGGTGAAAAACAT |
| MdBT4 (BD)-3 | TCACTGCCACAAAGTGCTG |
| MdBT2 (1-297) (BD)-5 | GAATTCATGGAAGCTAATCCGACC |
| MdBT2 (1-297) (BD)-3 | CTGCAGGCAAAGAGGGACTCTGC |
| MdBT2 (1-195) (BD)-5 | GAATTCATGGAAGCTAATCCGACC |
| MdBT2 (1-195) (BD)-3 | CTGCAGCTTCTTCCTCGATTCAATT |
| MdBT2 (196-361) (BD)-5 | GAATTCGAAAACGTGGTGGACGTGC |
| MdBT2 (196-361) (BD)-3 | CTGCAGCAATCTGAAGCTTCTAAT |
| MdGRF11 (RT)-5 | CTTGACGTGGAGCTCACTGT |
| MdGRF11 (RT)-3 | TTCAAAAGCAGTGCACGACG |
| MdBT2 (RT)-5 | ACTCTCAGTAGCCGTCTCC |
| MdBT2 (RT)-3 | GCTCCAAAGCAAGAAGAAGG |
| MdGRF4 (RT)-5 | CCGAGCAAGCCGAGCGTTAC |
| MdGRF4(RT)-3 | AGATTCCGCTCCTCTACGGTGAG |
| MdGRF6 (RT)-5 | TTGAGCAGAAGGAGGAGAGCAGAG |
| MdGRF6 (RT)-3 | CGGCAGATGAGGCAGATGGAATG |
| MdGRF18 (RT)-5 | TTGAGCAGAAGGAGGAGAGCAGAG |
| MdGRF18 (RT)-3 | ACTCGGCGGAAGAGGCAGAC |
| 18s-5 | ACACGGGGAGGTAGTGACAA |
| 18s-3 | CCTCCAATGGATCCTCGTTA |
| MdDFR (RT)-5 | GTTGAGGGAGATAGGGTTTGAG |
| MdDFR (RT)-3 | GGTAAATGTAAAACAATAGAGAGG |
| MdF3H (RT)-5 | GCCGATCACCTACACCGAG |
| MdF3H (RT)-3 | GTACAAGAAGTGGGAAGGC |
| MdANS (RT)-5 | GGAGAAGATCATCCTTAAGCCA |
| MdANS (RT)-3 | CTAAGATATATCATACCAACTATGCC |
| MdUF3GT (RT)-5 | GGAAGTGGTTTTGTCGCCTG |
| MdUF3GT (RT)-3 | CATTATTATTGAGCAACGAACAGC |
| GST-MdBT2-5 | GGGTTTCCGAAGGAGTTC |
| GST-MdBT2-3 | GACAACCTTGCCAATAATG |
| His-MdGRF11-5 | GGATCCATGGCTTCCCCCAAAG |
| His-MdGRF11 -3 | GTCGACCTCTGCATCTTCACCTC |
| MdBT2-YFP^N^-5 | TCTAGAATGGAAGCTAATCCGACC |
| MdBT2-YFP^N^-3 | CCCGGGCAATCTGAAGCTTCTAAT |
| MdGRF11-YFP^C^-5 | GTCGACATGGCTTCCCCCAAAG |
| MdGRF11-YFP^C^-3 | CCCGGGCTCTGCATCTTCACC |
| MdGRF11 (pCXSN)-5 | AatggcttcccccaaagagAGAGA |
| MdGRF11 (pCXSN)-3 | CTCTGCATCTTCACCTCCAGGTTTA |
| MdBT2 (pCXSN)-5 | AATGGAAGCTAATCCGACCG |
| MdBT2 (pCXSN)-3 | CAATCTGAAGCTTCTAATTCCATG |
| MdBT2-Anti (pCXSN)-5 | CTGAAGTTGGTCGCTAATCACT |
| MdBT2-Anti (pCXSN)-3 | CTTCTCCTAATTCTTCCTCCCTC |
| pIR-MdGRF11-5 | GTCGACATGGCTTCCCCCAAAG |
| pIR-MdGRF11 -3 | TCTAGACTCTGCATCTTCACCTCCA |
| TRV-MdGRF11-5 | TCTAGAGAAATGGTGGAGGCGATG |
| TRV-MdGRF11 -3 | CCCGGGCCTCATGATGTCGCTGCA |
| pIR-MdBT2-5 | CCTCGAGATGGAAGCTAATCCGAC |
| pIR-MdBT2-3 | CTCTAGACAATCTGAAGCTTCTAAT |
| TRV-MdBT2-5 | GAATTCTGAAGTTGGTCGCTAATCACT |
| TRV-MdBT2-3 | TCTAGACTTCTCCTAATTCTTCCTCCCTC |
